# A Comparative Multicenter Cross-Sectional Study on Hypoglycemia in Young Adults and Older Adults With Type 2 Diabetes Mellitus in an Outpatient Setting in Sri Lanka

**DOI:** 10.1155/jdr/4412070

**Published:** 2025-08-22

**Authors:** Priyamali Thusharika Jayasekera, Thamarasi Senaratne, Dilusha Lamabadusuriya, Harsha Sathischandra, Anne Thushara Matthias, Pubudu Jayasekera, Mettananda Herath, Warsha Dilani De Zoysa, Ganaka Senaratne, Manoji Pathirage, Kumuduni Jayasinghe, Shashi Karunaratne, Ponnuthurai Sudarshan, Vathulan Sujanitha, Nalayni Rajaratnam, Deeptha Wickramaratne, Maheshwaran Umakanth, Bandusiri Ratnayake, Lalith Rasnayake, Roshan Liyanage, Jagath Pushpakumara, Thushari Wanigarathne, Prasad Siriwardhane, Yapa Udaya Kumara, Shanil Kuruppu, Deepthi Edirisinghe, Namal Wijesinghe

**Affiliations:** ^1^Department of Medicine, Faculty of Medicine, General Sir John Kotelawala Defence University, Colombo, Sri Lanka; ^2^Faculty of Allied Health Sciences, General Sir John Kotelawala Defence University, Colombo, Sri Lanka; ^3^Ministry of Health, Nutrition and Indigenous Medicine, Colombo, Sri Lanka; ^4^Faculty of Medicine, University of Sri Jayewardenepura, Colombo, Sri Lanka; ^5^Faculty of Medicine, University of Ruhuna, Galle, Sri Lanka; ^6^Faculty of Medicine, University of Peradeniya, Peradeniya, Sri Lanka; ^7^Faculty of Medicine, University of Jaffna, Jaffna, Sri Lanka; ^8^Faculty of Medicine, Eastern University of Sri Lanka, Batticaloa, Sri Lanka; ^9^University Hospital Kotelawala Defence University, Colombo, Sri Lanka

**Keywords:** driving, frailty, hypoglycemia, Sri Lanka, young and older adults

## Abstract

**Background:** Hypoglycemia has been an often-neglected complication of diabetes therapy. Mild hypoglycemia reduces quality of life, while severe hypoglycemia is life-threatening and can precipitate major cardiovascular and cerebrovascular events.

**Methods:** A multicenter cross-sectional study was conducted on hypoglycemia among adults with diabetes attending 19 medical clinics in government hospitals in Sri Lanka using an interviewer-administered questionnaire.

**Results:** There were 2005 participants, and 1110 (55.4%) were < 65 years of age and 895 (44.6%) were > 65 years of age; the mean age was 62.12 ± 11.94 years (58.1% female). The median duration of diabetes was 8 (IQR11) years. Among them, 808 (43%), 757 (37.8%), and 376 (18.8%) had neuropathy, retinopathy, and nephropathy, respectively, while 415 (20.7%), 50 (2.5%), and 22 (1%) had ischemic heart disease, strokes, and peripheral vascular disease, respectively. One thousand three hundred forty-nine (67.3%) experienced at least one episode of hypoglycemia, and 462 (34.2%) had hospital admissions (9 (0.7%) intensive care admissions) over the past year. Older adults (*n* = 584) experienced significantly more symptomatic hypoglycemia compared to the younger population (*p* ≤ 0.001). Their mean CBS during hypoglycemic episodes was 57.04 ± 18.15 mg/dL. Among them, 1552 (77.4%) were on oral hypoglycemic medications, 453 (22.6%) were on insulin, and 126 (6.3%) were on both. The most typical reasons for hypoglycemia were skipping meals while taking regular medications (511, 37.9%), consumption of sugar-reducing native food items (203, 15%) and taking higher doses of insulin (112, 8.3%) and oral medication (74, 5.5%) than prescribed. To self-manage hypoglycemia, 1070 (79.3%) took sugary drinks, food, or glucose, and 279 (20.7%) did not do anything. There were 304 (34%) frail older adults, and 238 (78.3%) got hypoglycemia. They were taking similar prescriptions as young adults. They displayed significant hypoglycemic symptoms such as dizziness, irritability, nausea, speech impediment, and blurred vision compared to nonfrail elders (*p* ≤ 0.01). There were 485 diabetic patients who either drove or rode in a vehicle; 51 (10.5%) of them had experienced hypoglycemia during driving or riding.

**Conclusion:** Hypoglycemia is a significant issue which needs to be addressed. There is no difference in prescription medication in age categories and frail patients. Driving and hypoglycemia are also a concerning issue. Patients need advice on the prevention and treatment of hypoglycemia.

**Summary**

• Why did we undertake this study? There is a huge gap in studies on hypoglycemia in Sri Lanka as well as the region.
• What is the specific question(s) we wanted to answer? Why do people get hypoglycemia; are there age differences in symptoms and how they act; how many are aware of things to do; how many frail patients are getting hypoglycemia and their treatment regime and safety of driving and hypoglycemia?
• What did we find? Hypoglycemia is a significant issue that needs to be addressed. There is no difference in prescription medication in age categories and frail patients. Driving and hypoglycemia are also concerning issues. Patients need advice on the prevention and treatment of hypoglycemia.
• What are the implications of our findings? Knowledge of diabetes and hypoglycemia needs to be improved. Treatment should be individualized, and medical officers should be trained to tailor the diabetic medication prescription. Driving license authorities should be given guidelines on hypoglycemia and driving.

## 1. Introduction

Hypoglycemia is a condition of reduced blood glucose levels below the normal range. According to the latest recommendations of the American Diabetes Association, hypoglycemia is recognized as a decrease in blood glucose below 70 mg/dL (3.9 mmol/L). Further, according to the Ademolus classification of hypoglycemia (ACH), hypoglycemia can be subcategorized as Grade 1 (mild) 55–70 mg/dL, Grade 2 (moderate) 40–54.9 mg/dL, Grade 3 (severe) 10–39.9 g/dL, and Grade 4 (very severe) < 10 mg/dL [1]. Hypoglycemia is one of the most common acute complications of diabetes therapy. Drug-induced hypoglycemia may occur in any type of diabetes, mainly as a side effect of treatment with insulin or sulfonylureas. Severe hypoglycemia is characterized as an occurrence linked to severe cognitive impairment (such as coma and convulsions) that necessitates the active administration of carbohydrates, glucagon, or other corrective measures by a third party to aid the patient's recovery rather than treating himself [2]. Even though plasma glucose concentrations might not be accessible during an incident, the neurological recovery when plasma glucose levels return to normal proves that low plasma glucose induced the event [3]. Recurrent severe hypoglycemia is defined as two or more hypoglycemia events in the last 12 months [4].

Symptoms associated with hypoglycemia are sweating, tremors, hunger, palpitations, mood changes, and headache. Moderate hypoglycemia is related to dysfunction of the central nervous system (CNS). It may include dizziness, anxiety, confusion, ataxia, and blurred vision. Blood glucose below 45 mg/dL (2.5 mmol/L) may cause profound disturbances of the CNS with loss of consciousness and generalized convulsions [5]. A hypoglycemic coma is a medical emergency usually associated with a blood glucose level of around 20 mg/dL (1.1 mmol/L). Prolonged severe hypoglycemia may result in permanent neurological disorders. Loss of consciousness may be accompanied by bilateral plantar reflexes, muscle tension, and hypothermia [5]. The association between severe hypoglycemia and morbidity is likely exacerbated by frailty [4, 6]. Reducing severe hypoglycemia in patients with frailty may significantly reduce morbidity and improve quality of life.

Ageing impairs an effective counterregulatory response to hypoglycemia, which is added to the autonomic compromise present in all patients with diabetes. The brain depends upon renal, hepatic, and endocrine regulation of glucose levels. Ageing physiology also changes the pharmacokinetics of oral medications and insulin, particularly drug absorption, distribution, and renal elimination [7].

Symptoms of hypoglycemia change with age, and they are often atypical in elderly individuals. Older people are also more susceptible to iatrogenic hypoglycemia. Therefore, the identification and treatment of hypoglycemia tend to be delayed, leading to increasing disease severity. The ADVANCE (Action in Diabetes and Vascular Disease: Preterax and Diamicron MR Controlled Evaluation) trial reported that patients with Type 2 diabetes who experience severe hypoglycemia are at higher risk of a significant macrovascular event or death over the subsequent 12 months [8].

In this study, we defined individuals 65 years and older as elderly. This is mainly due to the retirement age and the working population being up to the age of 65 years in Sri Lanka. Regarding cardiovascular diseases, 75 years is often cited as the beginning of old age despite such individual variation. Likewise, 85 years is frequently used as a practical threshold to classify very old age. These cut-offs presume that by 75 and 85, most adults have sustained sufficient ageing changes to exhibit clinically relevant differences in physiology, organ function, and reserve [9, 10]. The age category in older people is taken according to this study.

## 2. Methodology

**Figure 1:**
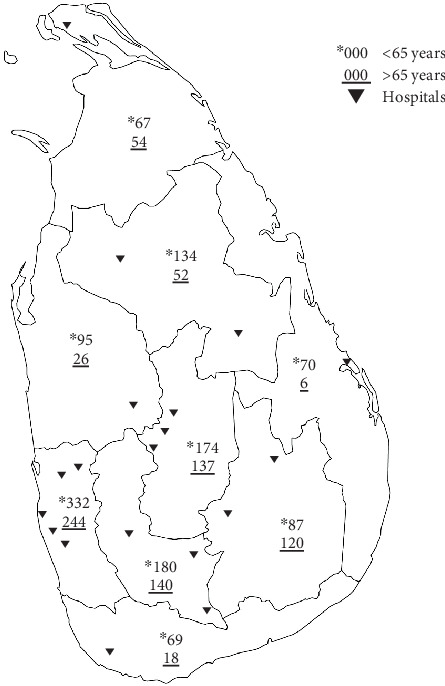
Population distribution in hospitals among Sri Lanka.

This multicenter comparative cross-sectional study was conducted in 19 consultant-based, rural and urban government hospitals in Sri Lanka (Figure 1). Patients aged 18 years and above (young and older adults) who are undergoing outpatient follow-up in diabetic clinics for Type 2 diabetes mellitus in Sri Lanka were included in the study. The cut-off age of 65 years and above was considered as older adults. Patients with decisional impairment with no legally authorized representative, cognitive impairment, and prisoners were excluded. Fasting blood sugar (FBS) of 126 mg/dL and HbA1C < 7% were taken as reasonable control. Hypoglycemia was diagnosed by symptoms and capillary blood sugar (CBS) < 70 mg/dL and, when the blood glucose was not available, improvement of symptoms after eating or drinking sugar-containing food. Further, hypoglycemia, with known CBS, was subcategorized according to the ACH [1].

The interviewer administered a questionnaire based on demographic data and hypoglycemic symptoms from the American Diabetes Association guidelines; it was administered by a medical officer trained by the respective coinvestigator. The face and content validity of the questionnaire were tested by two specialists in internal medicine who were not involved with the study. A pilot study was conducted in different hospitals before the study was initiated. Frailty was assessed using the Prisma 7 questionnaire, which is validated by the author and is in the process of publication. MMSE [11] and MOCA [12] Sinhala versions were used to screen the dementia patients.

## 3. Objective

### 3.1. General Objective

This study was aimed at finding the prevalence of hypoglycemia and evaluating the risk factors for developing hypoglycemia in diabetic patients undergoing outpatient treatment in Sri Lanka, with a particular emphasis on patient demographics, risk factors, clinical presentation, and treatment in the two main groups (young [< 65 years of age] and old [> 65 years of age]). Additionally, we examined the hypoglycemic characteristics of young (less than 65 years old) and old (more than 65 years old), as well as among the elderly group's subcategories of the old category (youngest old [65–74 years old], middle old [75–84 years old], and oldest old [> 85 years old]) [13].

Furthermore, we compared the incidence of hypoglycemia and frailty in older adults.

### 3.2. Sampling Method

Hospitals were selected using stratified random sampling, representing each province around the country. Simple random sampling was applied to recruit patients in each hospital.

### 3.3. Study Setting

The study setting was medical clinics in tertiary and secondary care government hospitals conducted by specialists in internal medicine in Sri Lanka. There were 19 hospitals selected using stratified random sampling (Figure 1and Table 1).

### 3.4. Statistical Analysis

The quantitative analysis of collected data was performed using IBM SPSS Software Version 25. Descriptive statistical analysis of the demographic data was performed to identify the characteristics of the study sample. An independent sample *t*-test was used to calculate the association between demographic factors and two groups, such as young and older adults. The association between other demographic factors and scores of three or more variables was analyzed using a one-way ANOVA test. The post hoc comparison using the Bonferroni test was performed to determine the differences within the groups. The limit for statistically significant differences was set at *p* < 0.05.

## 4. Results

### 4.1. Demographic and Basic Information

There were 2005 patients with Type 2 diabetes in the study, while 1110 (55.4%) were < 65 years of age and 895 (44.6%) were > 65 years of age, with a mean age of 62.12 ± 11.94 years. The majority were females (58.1%). Their mean body mass index (BMI) was 24.6 kg/m^2^ (SD 4.68) (25 kg/m^2^ [SD 4.9] younger, 24.1 kg/m^2^ [SD 4.4] older adults). These participants were from 19 diabetic clinics in the country, representing both rural and urban areas in all nine provinces of Sri Lanka (Table 1 and Figure 1).

Their median duration of diabetes was 8 years (IQR 11) (in young adults, 6 years [IQR 9], and older adults, 10 years [IQR 15]). Of them, 512 (25.5%) are currently employed, and 484 (24.1%) are driving or riding a vehicle. Except for 164 (8.2%) who did not have formal education, the rest had some form of education (Table 1). Their drug compliance, which was self-reported by the participants, was good, as 1583 (79%) took regular medications, while 422 (21%) sometimes skipped medications.

Only 491 (24.5%) had glucometers at home, and 34% were from the Western province. Eight hundred ninety-five (44.6%) had a normal FBS level (≤ 121 mg/dL) recorded during the previous month, and 826 (41.2%) had a value higher than expected. However, 284 (14.2%) had no FBS during the last 3 months.

Among them, 1552 (74.4%) were on oral hypoglycemics, 1327 (66.2%) on sulfonylurea (1306 [98.4%] gliclazide, 11 [0.8%] glibenclamide, 2 [0.15%] glimepiride, and 9 [0.7%] tolbutamide), 1468 (73.2%) on metformin, 557 (35.8%) on DPP4 inhibitors (549 sitagliptin, 8 linagliptin), 32 (1.6%) on pioglitazone, 55 (2.7%) on SGLT2 inhibitor, and 453 (22.6%) on insulins. Half of the participants (1031, 51.4%) were taking both metformin and sulfonylurea. Only 126 (6.3%) were on both oral hypoglycemics and insulin. Further, 610 (68.2%) elderly were on metformin, 602 (67.3%) were on sulfonylurea, 191 (21.3%) were on insulin, and 72 (8%) were on both oral hypoglycemics and insulin. Significant use of metformin is observed in older individuals (*p* ≤ 0.001).

A lipid profile was available in 272 (13.6%) patients within the last 3 months, with a mean total cholesterol of 179 (SD 55.4) and LDL 104 (SD 50). HbA1c for the previous 3 months was available only in 207 patients, and the mean was 8.82, SD 15 {median 7.1 (IQR 2.4)}.

The majority of diabetic patients suffered from hypertension, 1336 (66.6%) and 717 (53.7%) had uncontrolled hypertension (< 65 years blood pressure > 140/90 mmHg, > 65 years systolic blood pressure > 150 mmHg were taken as high blood pressure). The second most common was dyslipidemia, 1120 (55.9%); 378 had various other coexisting diseases (84 (4.2%) chronic kidney disease, 57 (2.9%) asthma, and 43 (2.1%) hypothyroidism).

When considering the diabetic microvascular complications, 757 (37.8%) had retinopathy, 808 (40.3%) had neuropathy, and 376 (18.8%) had nephropathy; 568 (28.3%) had cataract surgeries. Macrovascular complications were 415 (20.7%) ischemic heart disease, 50 (2.5%) cerebrovascular accidents, and 22 (1%) peripheral vascular disease.

There is a strong and statistically significant association between hypoglycemia and microvascular complications (*p* ≤ 0.001) (OR, 0.527 [95% CI 0.436–0.637]) but not with the macrovascular complications (OR, 1.301 [95% CI 0.585–2.892]). But microvascular (OR, 1.056 [95% CI 0.662–1.686]) or macrovascular (OR, 1.44 [95% CI 0.662–1.686]) involvement does not significantly change the risk of being in severe hypoglycemia.

Many had autonomic complications, such as 440 (21.9%) had postural giddiness, 125 (6.2%) had gustatory sweating, and 84 (10%) suffered from impotence.

In addition to the above complications, 237 (11.8%) had nail/toe fungal infections, 60 (3%) had tinea infections, and 161 (8%) had periodontitis. In addition to diabetic-related symptoms, many suffer from nonspecific symptoms such as bilateral ankle edema, constipation, and backache, which affect older adults significantly (Table 2).

### 4.2. Hypoglycemia

Out of the population of 2005, 1349 (67.3%) experienced at least a single episode of hypoglycemia over the last year. Six hundred forty-seven older adults had significantly more symptomatic hypoglycemia than the younger population (*p* ≤ 0.001). Furthermore, 462 (34.2%) had hospital admissions, with 9 (0.7%) intensive care admissions. However, only 665 can recall the CBS during the hypoglycemic episode (103 had CBS > 70 mg/dL). Their mean CBS during hypoglycemic episodes was 57.04 ± 18.15 mg/dL. According to the Ademolus classification, 294 (52.3%), 169 (30.1%), 97 (17.3%), and 2 (0.4%) had mild, moderate, severe, and very severe hypoglycemia, respectively (Table 3).

The most familiar hypoglycemic symptoms were cold sweat (1013, 75.1%), dizziness (911, 67.5%), and excessive tiredness (899, 66.6%). The most typical reason for hypoglycemia was skipping meals while taking regular medications (511, 37.9%), consumption of the sugar-reducing native food items (203, 15%), taking higher doses of insulin (112, 8.3%) and higher doses of oral medication (74, 5.5%) than prescribed, intercurrent illness (61, 3%), concomitant administration of traditional medicine (39, 3%), and alcohol (10, 0.5%). Five hundred ninety-two (29.5%) could not recognize the specific reason for hypoglycemia, and 391 (29%) thought their given drugs were “too strong” but continued to take the same dose despite hypoglycemia.

To self-manage hypoglycemia, 1070 (79.3%) took sugary drinks, food, or glucose, and 279 (20.7%) did not do anything, which was concerning. Furthermore, 226 (16.7%) omitted the next medication dose to prevent further hypoglycemia, whereas 339 (25.1%) continued to take the next dose of the medication. However, following a single episode, 405 (20%) always carry a sweet in case of hypoglycemia.

### 4.3. Hypoglycemia: Driving and Riding

There were 485 diabetic patients (100 of whom were over 65 years of age) who drove or rode a vehicle. There were 217 (44.7%) drivers and 268 (55.2%) riders. A total of 323 (66.6%) had experienced at least one episode of hypoglycemia during the last year, but only 51 (10.5%) had experienced hypoglycemia while driving or riding. Fourteen (27.4%) had hospital admissions. All of them recovered and were discharged within 24–48 h. The commonest symptoms were dizziness {189 (39%)}, sweating {226 (46.6%)}, and blurred vision {154 (31.8%)}. The majority of them were either on sulfonylurea {285 (58.8%)} or insulin {98 (20.2%)}. Five (1%) had hypoglycemia following alcohol consumption. None had accidents while driving or riding.

### 4.4. Hypoglycemia in Young and Elderly

Despite age differences, both age groups experienced several similar hypoglycemic symptoms, as explained. The elderly experienced more hypoglycemia than the young. Symptoms of cold sweat, cognitive dysfunction, and lethargy were significantly (*p* ≤ 0.001) more common in the elderly than in the young. Actions taken for hypoglycemia in both young and old were the same, except that older people always carried a sweet with them after experiencing a single episode of hypoglycemia (*p* ≤ 0.0001).

### 4.5. Frailty and Hypoglycemia

There were 304 (34%) frail older adults and 238 (78.3%) got hypoglycemia. There were only 17 (2%) dementia patients (according to MMSE and MOCA) who complained of memory loss. One hundred twenty-six (41.4%) had normal FBSs, whereas 114 (37.5%) had high blood sugar levels in routine clinic visits. Many (183, 60.2%) needed walking aids (125 canes, 30 walkers, 26 wheelchairs, one rollator, and one crutch). Most were on oral hypoglycemic medications (184 [60.5%] on metformin, 196 [64.6%] on sulfonylurea, 131 [43.1%] on metformin and sulfonylurea, 110 [36.2%] on a single drug, 75 [24.7%] on insulin, and 22 [7.2%] on both oral hypoglycemics and insulin). In older adults, a higher proportion of frail individuals were on metformin compared to nonfrail individuals (*p* ≤ 0.001). Their commonest hypoglycemic symptoms were dizziness and blurred vision. They displayed significant hypoglycemic symptoms such as dizziness, irritability, nausea, speech impediment, and blurred vision compared to nonfrail elders (*p* < 0.01) (Table 4). Frail adults take glucose or sugar to manage hypoglycemia more than others. No falls were reported secondary to hypoglycemia. Frail adults experienced a loss of hunger compared to nonfrail adults (*p* ≤ 0.05).

## 5. Discussion

Hypoglycemia in older diabetic patients is associated with severe adverse outcomes. This was illustrated in a meta-analysis published in 2020, which showed that hypoglycemia was associated with an increased likelihood of death (OR 2). There was also an association with dementia (OR 1.5), macrovascular complications pooled OR 1.81 (95% CI 1.70–1.94), and microvascular complications (two studies) pooled OR 1.77 (95% CI 1.49–2.10). The association between hypoglycemia and cardiovascular death (six studies) showed a pooled OR of 2.11 (95% CI 1.55–2.87). Six studies demonstrated an association between hypoglycemia and falls and fractures, with pooled ORs of 1.78 (95% CI 1.44–2.21) and 1.68 (95% CI 1.37–2.07), respectively [14]. These studies highlight how hypoglycemia must be avoided at all costs in this vulnerable population.

Severe single episodes of hypoglycemia may result in serious acute consequences such as seizure, coma, and cardiac arrhythmias, which were not reported in our study population. The outcome associated with these acute consequences can be particularly debilitating in older people who are at increased risk of injury, joint dislocation, and bone fractures [15]. However, there was no report of falls or associated injuries in our elderly population with hypoglycemia. In contrast to other studies, there were fewer hospital admissions and more self-management, possibly due to the lower severity.

In this study, we found that older adults had a longer duration of diabetes and had a significantly higher incidence of hypoglycemia (*p* ≤ 0.0001). A similar finding was reported in a study conducted in Sri Lanka, which showed that patients who experienced hypoglycemic episodes of any severity had a higher mean age (*p* ≤ 0.006) and a higher mean duration of diabetes (*p* ≤ 0.001) compared to those without [16]. Rising trends in age and duration of diabetes were observed with increasing severity of the episodes.

Most patients with hypoglycemia in our study were on sulfonylureas, which raise insulin levels independently of blood glucose, thereby increasing the risk of hypoglycemia. This finding is similar to that of the UKPDS [16–18], which identified secretagogues and exogenous insulin as the primary causes of hypoglycemia. Both young and older adults had similar prescribing patterns of oral hypoglycemic drugs and insulins, except that the frail elderly were prescribed metformin more than others, which is significant. A similar prescribing pattern was described in a study done in rural Sri Lanka. This shows that most patients depend on free medications provided by the government [19]. Prescribing metformin in a frail adult may affect their quality of life as it causes nausea and, subsequently, anorexia, which was found to be a significant symptom in this study. However, except for loss of hunger, which is more common in frail adults, we did not inquire about other side effects of medications in this study.

A significant percentage of older adults suffer from retinopathy, nephropathy, neuropathy, postural giddiness, and macrovascular complications (IHD, PVD, and stroke). These findings may be correlated with age and the duration of diabetes. However, both age categories suffered equally from impotence. This finding is alarming, though the duration of diabetes is shorter in the younger population; their poor glycemic control may have contributed to that finding. The majority had coexistent hypertension, whereas a significantly higher proportion of older adults were hypertensive. The incidence of dyslipidemia was the same in both age groups. In the elderly, people with retinopathy and ischemic heart disease had a significantly lower incidence of reported hypoglycemia. This may reflect that they may be more knowledgeable and careful with their disease after having such complications. There is no higher incidence of hypoglycemia in the elderly with dementia. Anyway, in our population, only 17 had dementia. This is because people with dementia may not accurately represent the findings of this study.

We found that 78% of frail older adults had suffered from hypoglycemia. Recurrent episodes of less severe hypoglycemia are associated with significant chronic consequences leading to physical and cognitive dysfunction and, eventually, frailty and disability [3]. The less severe hypoglycemia, therefore, has a substantial long-term impact on older people and is of particular importance as it may not present as readily as the more severe hypoglycemia. Lethargy was a significant hypoglycemic symptom in older people. Frailty and hypoglycemia are closely interlinked. Recurrent hypoglycemia can cause frailty; a frail person is more likely to get hypoglycemia.

A 29.7% of people did not take any action after an episode of hypoglycemia and did not even omit the next dose of medication. The level of education did not have a significant impact on the management of hypoglycemia.

One of the main limitations of the study was that we relied on self-reporting of symptoms of hypoglycemia to diagnose hypoglycemic episodes. There can also be a recall bias. As there are many difficulties in recognizing hypoglycemia in older people due to nonspecific symptoms, misdiagnosis of hypoglycemia as a stroke, vertigo, or dementia-related symptoms can occur. Hypoglycemia can also have atypical presentations in older people, such as hyperactive or hypoactive delirium. Additionally, patients with dementia are unable to communicate their feelings or symptoms adequately. This study was conducted in government hospitals, where patients receive free medications, and therefore, it may not accurately represent patients who purchase medication from the private sector. Most do not have glucometers, so we cannot find the exact CBS levels. The availability of HbA1C is limited, as it is only freely available in some government hospitals. These factors may have resulted in an underestimation of the incidence of hypoglycemia in the elderly subgroup.

## 6. Conclusion

To our knowledge, this study is the first in Sri Lanka to study the relationship between frailty and hypoglycemia. We found that there was a high incidence of hypoglycemia in older diabetic patients with frailty. However, there was no change to the prescribing pattern of oral hypoglycemic medication in the older age group, except that they were given metformin rather than younger patients. This may be a reflection that the same medications are carried out without altering the current status of life. Therefore, targets in frail older people with diabetes should focus on short-term day-to-day blood glucose levels to avoid hypoglycemia rather than targeting diabetes care around a long-term HbA1c strategy. Prescriptions and glycemic targets should be tailor-made for each individual when it comes to the elderly and frail. This principle should be disseminated more widely among medical professionals. Adequate knowledge of diabetes for all patients and advising them on what to do and what not to do in hypoglycemia will help them. This also discusses driving and hypoglycemia, although not all individuals are professional drivers. There was a reasonable number of people who felt hypoglycemic while driving, which is also alarming. It is essential to inform medical professionals about this knowledge gap in diabetes awareness among patients and take necessary steps to empower them with the necessary information. Furthermore, guidelines for diabetic individuals and driving need to be established in consideration of road safety.

## Figures and Tables

**Table 1:** Demographic data.

**Distribution of the participants in Sri Lanka (** **n** = 2005**)**		

Province	< 65 years	> 65 years
Western	332	244
Northern	67	54
North Central	134	52
Central	174	137
Northwestern	95	26
Eastern	70	6
Uva	87	120
Sabaragamuwa	180	140
Southern	69	18
Total	1208	797
Level of education	Number of patients	
No formal education	164 (8.2%)	
Studied up to Grade 5	291 (14.5%)	
Studied up to Grade 8	408 (20.3%)	
Studied up to ordinary level	734 (36.6%)	
Studied up to advanced level	346 (17.3%)	
Graduates	57 (2.8%)	
Post graduate qualifications	5 (0.24%)	
Employment status		
Currently employed	444 (22.1%)	
Unemployed	616 (30.7%)	
Retired	148 (7.4%)	

Driving or riding a vehicle (*n* = 485)		
Category of vehicle	Drivers *n* = 217	Hypoglycemia while driving/riding

Light vehicle	162	20
Heavy vehicle	36	6
Three wheelers	10	2
Not specified	08	0
	Riders *n* = 268	
Motor bikes	200	14
Pedal cyclist	68	9

**Table 2:** Associated diseases and complications.

**Medical condition**	**Y** **o** **u** **n** **g** **e** **r** < 65** (n** = 1110**)**	**O** **l** **d** **e** **r** > 65** (n** = 895**)**	**Significance**
Hypertension	662	673	*p* ≤ 0.0001
Dyslipidemia	609	510	*p* ≤ 0.225

*Nonspecific signs and symptoms*			
Bilateral ankle edema	176	194	*p* ≤ 0.001
Constipation	323	297	*p* ≤ 0.137
Backache	362	346	*p* ≤ 0.018
Leg cramps	332	281	*p* ≤ 0.835
Dementia (< 25 MMSE^a^ or < 26 MOCA^a^)	0	17	

*Hypoglycemic symptoms*			
Hypoglycemic attacks	702	647	*p* ≤ 0.0001
Cold sweat	551	462	*p* ≤ 0.022
Lethargy	298	365	*p* ≤ 0.001
Speech impediment	180	194	*p* ≤ 0.306
Cognitive impairment	135	214	*p* ≤ 0.0001

*Microvascular complications*			
Retinopathy	344	413	*p* ≤ 0.0001
Neuropathy	402	405	*p* ≤ 0.0001
Nephropathy	172	204	*p* ≤ 0.0001

*Macrovascular complications*			
Ischemic heart disease	181	234	*p* ≤ 0.00001
Peripheral vascular disease	1	25	*p* ≤ 0.00001
Stroke	0	50	*p* ≤ 0.00001

*Autonomic complications*			
Impotence	47	37	*p* ≤ 0.958
Gustatory sweating	70	55	*p* ≤ 0.943
Postural giddiness	218	222	*p* ≤ 0.010

*Other*			
Cataract	228	340	*p* ≤ 0.001
Nail/toe web fungal infections	116	121	*p* ≤ 0.026
Tinea infection (generalized)	33	27	*p* ≤ 0.282
Periodontitis	77	84	*p* ≤ 0.036

Abbreviations: MMSE, Mini-Mental State Examination; MOCA, Montreal Cognitive Assessment.

^a^Sinhala version.

**Table 3:** Ademolus classification of hypoglycemia.

**Age category**	**Mild**		**Moderate**		**Severe**		**Very severe**	
18–65 years *n* = 306	165	53.9%	95	31%	46	15%	0	0
> 65 years *n* = 255	128	50.2%	74	29%	51	20%	2	0.8%

**Table 4:** Frailty and hypoglycemic symptoms.

**Age (years)** **(Frail)**	**Frail elders (** **n** = 304** hypoglycemia in 238)** **Nonfrail elders (n** = 581** hypoglycemia in 409)**		

65–74	182		
75–84	87		
> 85	35		

**Hypoglycemic symptom**	**Frail (** **n** = 238**)**	**Nonfrail (** **n** = 409**)**	**Significance**

Cold sweat	177	317	*p* ≤ 0.336
Dizziness/light headedness	**174**	**258**	**p** ≤ 0.025
Fasting irritation	**65**	**66**	**p** ≤ 0.0001
Nausea	**68**	**76**	**p** ≤ 0.011
Palpitations	88	150	*p* ≤ 0.921
Headache	85	129	*p* ≤ 0.220
Speech impediment	**90**	**97**	**p** ≤ 0.0001
Hunger	82	154	*p* ≤ 0.402
Weakness/fatigue/feeling languid	158	256	*p* ≤ 0.317
Lethargy	137	217	*p* ≤ 0.256
Blurred vision	**122**	**163**	**p** ≤ 0.006
Hyperphagia	54	72	*p* ≤ 0.166
Tremulousness	117	164	*p* ≤ 0.057
Frequent yawning	44	73	*p* ≤ 0.831
Coldness	63	84	*p* ≤ 0.080
Asymptomatic	04	11	*p* ≤ 0.413
Cognitive impairment	77	101	*p* ≤ 0.074

*Note:* Bold ones are the statistical significance where *p* < 0.05.

## Data Availability

Data is available with the corresponding author on request.

## Nomenclature

FBS: fasting blood sugar
CBS: capillary blood sugar
HbA1C: glycated hemoglobin
MMSE: Mini-Mental State Examination
MOCA: Montreal Cognitive Assessment
IHD: ischemic heart disease
PVD: peripheral vascular disease
UKPDS: UK Prospective Diabetes Study
